# Formal Comment; Tracing the source of infection of cystic and alveolar echinococcosis, neglected parasitic infections with long latency: The shaky road of “evidence” gathering

**DOI:** 10.1371/journal.pntd.0009296

**Published:** 2021-03-30

**Authors:** Paul R. Torgerson, Lucy J. Robertson

**Affiliations:** 1 Section of Epidemiology, Vetsuisse Faculty, University of Zürich, Switzerland; 2 Parasitology, Department of Paraclinical Sciences, Faculty of Veterinary Medicine, Norwegian University of Life Sciences, Oslo, Norway; McGill University, CANADA

In their viewpoint article [1], Cassulli and Tamorozzi stated that three “similar” [2–4] systematic reviews and meta-analyses on echinococcosis gave different, and maybe conflicting, results. However, although these three studies seem superficially similar, closer consideration of these articles would reveal that this is not the case. The study of Conraths et al [3] was on human alveolar echinococcosis (AE) and that of Possenti et al [4] was on human cystic echinococcosis (CE). Both studies investigated potential risk factors for infection and undertook a meta-analysis of these factors. In contrast, the study of Torgerson et al [2] focused on both diseases. Thus, there are very important differences between the three studies. AE is caused by *Echinococcus multilocularis*, whereas CE is caused by *E*. *granulosus* sensu lato. These two parasites have significant differences in their ecologies, although for both, humans become infected as a spillover from their lifecycles. Whereas *E*. *multilocularis* has a largely wildlife lifecycle, usually cycling between foxes and small mammals, *E*. *granulosus* usually cycles between dogs and domestic livestock.

More importantly, however, in the studies of Conraths et al [3] and Possenti et al [4] only the odds ratios (OR) were analysed, whereas the study of Torgerson et al [2] focused on the attributable fraction (AF). The AF depends not only on the risk or OR, but also on the proportion of the population exposed to the risk factor. A risk factor may have a very high OR, but—should the proportion of the population exposed to the risk factor be low—then the AF will be low. Thus, it would not be surprising that a meta-analysis of OR would appear to produce different results to one that considered AF. A simple example can illustrate this difference. Imagine a cross-sectional study in which a population of 1000 people were examined by ultrasound. Of these, 100 had echinococcosis (10%). Of the population considered (1000 people), 10 owned dogs, and of the 10 dog owners, 9 had echinococcosis. Thus, the risk ratio for dog ownership is 9.79 and the OR is 88.91. Therefore, dog owners have a massively increased risk of echinococcosis compared with non-owners of dogs. BUT—of the 100 cases of echinococcosis, 91 were in people who did not own dogs. Thus, the AF to dog ownership is just 0.0808 or approximately 8.1%. Thus, being a dog owner has a high odds of being infected, but a low AF. This result is not conflicting, as it may seem, but a inevitable consequence of asking different questions of the same data.

There is a tendency to assume that because something is possible, then it must happen. For both AE and CE there is the theoretical possibility that food maybe contaminated with *Echinococcus* eggs resulting in the foodborne pathway of human infection. Because of this, there is a common tendency to state that food is a major pathway of human infection; this is perhaps also a result of several publications [5–7] that indicate that a substantial proportion of echinococcosis is foodborne. However, these reports are based on expert opinion rather than upon empirical evidence. The analysis of Torgerson et al [2] was an attempt to analyse the available evidence to estimate the proportion of disease that is attributable to food and to other potential pathways of infection. The studies of Conraths et al [3] and Possenti et al [4], however, investigated risk factors, and thus these studies were not asking the same research question and, importantly, not undertaking the same analysis of the data.

Torgerson et al [2] considered five main pathways of potential infection: fox contact, dog contact, contaminated food, contaminated water, and contaminated environment for AE; for CE the same pathways were considered, but excluding fox contact. This then brings in another important difference between these studies. In the study of Conraths et al [3], they also identified various characteristics of the people infected, such as gender, age, and ethnic group, as being risk factors for AE. However, these associated characteristics do not give any indication regarding the pathway of infection. For example, these factors do not tell us how an adult Tibetan woman became infected, merely that she was at a higher risk of infection. In our analysis [2], we would have assumed that the adult Tibetan woman had been infected through dog or fox contact, water, food, or environment. She may be at greater risk because she is the individual caring for the dog or the person collecting water, not directly because of being an adult Tibetan female. This is a fundamental and important difference between the analysis of Torgerson et al [2] and the other 2 studies [3–4].

In our opinion, it was reasonable to use a number of different “risk factors” as proxy for dog contact, such as feeding offal to dogs, as, essentially, the route of infection is contact with infected dogs [2]. It is also incorrect to suggest that the analysis used by Torgerson et al [2] did not consider a contaminated environment; transmission from a contaminated environment was assumed to be the residual AF when the other pathways had been considered, given that no other plausible pathways exist (i.e., the 4 (CE) or 5 (AE) pathways, when summed, should come to 1).

Casulli and Tamarozzi [1] made the comment “*Torgerson and colleagues found that foodborne attributable fractions (AF) for CE (AF 0*.*23*, *confidence interval (CI) 0*.*02 to 0*.*47)*, *for instance*, *were actually the same of the figures provided by the expert elicitation of the FERG (Foodborne Disease Burden Epidemiology Reference Group of the WHO) study (around AF 0*.*2*, *interquartile range (IQR) <0*.*05% to 50%)”*. This may suggest that the empirical evidence gives a similarly inaccurate AF as the expert opinion used in [6], possibly due to insufficient data. It also could be argued, from the alternative perspective, that using expert opinion came close to providing the correct answer. In this respect, the wisdom of the crowds is a well-known phenomenon. The crowd at a county fair accurately guessed the weight of an ox when their individual guesses were averaged (the average was closer to the ox’s true butchered weight, than the estimates of most crowd members) [8]. This implies that if you ask enough people to estimate an answer to a question, then the average of these answers should be close to the true value; perhaps more experts were needed to be asked to narrow the uncertainty. However, there is a contrasting example in which experts clearly gave the wrong answer when they indicated a substantial burden of foodborne pathogens was attributed to pork consumption in the Middle East [9].

We agree that there are issues related, inter alia, to recall bias because of the length of time between infection and clinical disease. This long incubation period makes these types of studies of AE and CE particularly challenging. Nevertheless, such data is all that we currently have available to address these important questions. Finally, it is true that the study of Torgerson et al [2] was more recent and did not access the same databases or have the same language restrictions. As a consequence, Torgerson et al [2] were able to locate more usable data, particularly from China. However, recognising the contributions of Conraths et al [3] and Possenti et al [4], we also cross-checked the bibliographies of these two articles during the preparation of our own to ensure that we had included all the data that had been used in previous studies.

Taking an overview, infected dogs are essential for onwards transmission of CE to humans. Whether this occurs through direct contact or through contamination of food, water, or the environment is, arguably, of secondary concern. To prevent the disease in humans, elimination of *E*. *granulosus* from the dog population is the essential step. This is less straightforward for AE. Where *E*. *multilocularis* is endemic there is usually a stable wildlife lifecycle. Thus for AE, knowledge of the source attribution is important for identifying the most appropriate measures to implement for the prevention of transmission to people.

In conclusion, the road of evidence gathering may be “somewhat” shaky. Nevertheless, when the different study designs, along with the more extensive literature that was located in the most recent of these three studies, are taken into account, it would, in our opinion, be premature to conclude that the evidence in these three studies is conflicting.
